# Assessment of Complement Cascade Components in Patients With Bipolar Disorder

**DOI:** 10.3389/fpsyt.2018.00614

**Published:** 2018-11-27

**Authors:** Artur Reginia, Jolanta Kucharska-Mazur, Marcin Jabłoński, Marta Budkowska, Barbara Dołȩgowska, Leszek Sagan, Błazej Misiak, Mariusz Z. Ratajczak, Janusz K. Rybakowski, Jerzy Samochowiec

**Affiliations:** ^1^Department of Psychiatry, Pomeranian Medical University, Szczecin, Poland; ^2^Department of Medical Analytics, Pomeranian Medical University, Szczecin, Poland; ^3^Department of Laboratory Medicine, Pomeranian Medical University, Szczecin, Poland; ^4^Department of Neurosurgery, Pomeranian Medical University, Szczecin, Poland; ^5^Department of Genetics, Wroclaw Medical University, Wrocław, Poland; ^6^Stem Cell Institute at James Graham Brown Cancer Center, University of Louisville, Louisville, KY, United States; ^7^Department of Adult Psychiatry, Poznan University of Medical Sciences, Poznan, Poland

**Keywords:** bipolar disorder, complement system, C3a, C5a, C5b-9

## Abstract

**Introduction:** The immune system is undoubtedly involved in the pathogenesis of various psychiatric disorders, such as schizophrenia, bipolar disorder, or depression. Although its role is not fully understood, it appears that this area of research can help to understand the etiology of mental illness. One of the components of the human immune system is the complement system, which forms a part of the innate immune response. Physiologically, except for its essential protective role, it is a vital element in the regeneration processes, including neurogenesis. To date, few studies have tried to clarify the role of the complement cascade in mental disorders.

**Materials and Methods:** We evaluated concentrations of C3a, C5a, and C5b-9 complement cascade components in the peripheral blood of 30 patients suffering from bipolar disorder (BD) for at least 10 years, in euthymia, who were not treated with lithium salts. In addition, we divided our study sample into BD type I (BD-I, 22 persons), and BD type II (BD-II, 8 patients). The control group consisted of 30 healthy volunteers matched for age, sex, BMI, and smoking habits.

**Results:** Compared to healthy controls, BD patients had elevated concentrations of all the investigated components. Furthermore, in patients with BD-II, we observed higher concentrations of C5b-9 as compared to patients with BD-I. However, there was a significant effect of BD diagnosis only on the levels of C3a and C5a but not on the level of C5b-9 after adjustment for potential confounding factors.

**Conclusions:** Increased concentrations of components C3a and C5a of the complement system in the investigated group as compared to healthy controls suggest involvement of the complement cascade in the pathogenesis of BD, and provides further evidence of immune system dysregulation in BD patients.

## Introduction

Despite enormous efforts of researchers, undeniable progress of knowledge and research opportunities provided by modern medicine, the etiology of BD is still not fully understood. The prevalence of BD has been estimated at over 1% of the general population, regardless of their origin, ethnicity, or socio-economic status (1). Proper diagnosis, especially in the early stages of the disease causes considerable difficulties (2). The disease itself, as well as its improper diagnosis or treatment lead to enormous social consequences and economic costs (3, 4).

Several lines of evidence indicate that BD has a multifactorial etiology, comprising both genetic (5, 6) and environmental factors (7, 8). The concept of viewing mental disorders as the consequences of aberrant immune-inflammatory processes has recently become the subject of numerous studies (9–11). It appears that what is observed in the course of BD are both the activation of inflammatory processes within the central nervous system and systemic inflammatory reactions (12, 13). Such arguments are supported by an increased activity of the hypothalamic-pituitary-adrenal axis (especially in the manic phase) and increased peripheral metabolism of cortisol (14). Moreover, it is hypothesized that exposure to certain infectious agents in the prenatal period may lead to the occurrence of BD (15), although results in this field remain somewhat inconclusive. Both exposure to infectious agents in the prenatal period and the aforementioned activation of the HPA axis may potentially affect the function of the immune system (16, 17).

*Post-mortem* studies indicate an increased expression of inflammatory markers and excitotoxicity in the frontal cortex of patients with BD compared to healthy controls (18–20). Examination of cerebrospinal fluid provides some further evidence of higher concentrations of interleukin-8 (IL-8) linked to the treatment of BD with lithium salts (21). Other studies demonstrate higher levels of monocyte chemoattractant protein 1 and chitinase-3-like protein 1 (22–24), and changes in concentrations of lymphocytes Th1, Th2, or cytokines. Some of these changes depend on the stage of the disease (25–27).

A growing body of evidence points to the fact that the activation of systemic inflammatory reactions occurs in the course of mood disorders, including BD (28, 29). Furthermore, there are increased levels of proinflammatory cytokines in the peripheral blood of patients with BD. While the findings are not conclusive, cytokine concentrations appear to vary depending on the stage of the disease and its subtype (30, 31). During euthymia there are increased concentrations of IL-10, TNF-α, and increased levels of neutrophils and monocytes (32). Epidemiological studies show higher comorbidity of BD and autoimmune diseases or metabolic disorders, whose pathogenesis is mediated by inflammatory processes (33–35).

The central nervous system was traditionally perceived as an immunologically privileged organ, but nevertheless there is some communication between the CNS neural tissue with the immune system (36, 37). In addition, leakages within the blood-brain barrier can occur during the periods of BD exacerbation, and thus a cross-talk between the central nervous system and the immune system may be facilitated (38, 39).

The complement system is involved in both immunological as well as regenerative processes. It consists of dozens of proteins produced mainly in the liver and in small amounts also by neurons, microglia, astrocytes and oligodendrocytes, and several cell receptors. In addition to participating in the immunological mechanisms, it plays an important role in processes such as: reducing inflammatory reaction, removal of apoptotic cells, angiogenesis, wound healing, repair processes and the mobilization of some types of stem cells. It is considered to play a role in the pathogenesis of neurodegenerative diseases (40–42). Complement component C3a affects neurogenesis, stimulates the differentiation of neural progenitor cells under hypoxic conditions. It also modulates astrocytes' response to ischaemia, increasing their ability to survive stress conditions associated with ischemia. During the development of nerve cells in fetal life, it acts as a chemoattractant for these cells (43–46). Soluble anaphylatoxins (C3a, C4a, and C5a) control local inflammatory response by activating and attracting leukocytes (47). The presence of the receptors C5aR C3aR on neurons can prevent their apoptosis, while sublithic levels of C5b-9 can protect oligodendrocytes from apoptosis. At the same time, it is known that the activated complement is involved in a process called synaptic elimination, it enhances the secretion of proinflammatory cytokines by glial cells and induces neuronal damage and death by C5b-9 (48). The C5a component has a neuroprotective effect on mature neurons (49).

Despite all past attempts at defining the role of the complement system in the etiology of BD remains uncliear. Certainly, it is involved in the neuroinflammation process (50). It is one of the many factors, whose interactions lead to the development of BD (51). The complement system is also believed to be one of the elements linking the theory of prenatal infection or hypersensitivity to gluten with the development of neuropsychiatric disorders (52).

The aim of this study was to evaluate alterations in the levels of complement components in patients with BD as well as to determine whether these alterations are related to psychopathological manifestation of BD.

## Materials and methods

### Participants

The study involved 30 unrelated patients suffering from BD for at least 10 years, not treated with lithium salts for at least 5 years prior to the study, due to the potential impact of lithium salts on inflammation and regeneration processes (53). A diagnosis of BD was established according to the ICD-10 criteria (54). At the time of the study all patients were in a stabilized mental state and met the criteria for BD remission. Exclusion criteria were presence of active substance dependence in the last 6 months (except for nicotine addiction); current or lifetime history of significant organic brain damage; cognitive impairment typical of dementia; serious somatic diseases, glucose intolerance, currently active inflammatory disease (exclusion based on the results of laboratory tests and physical examination); mental disorder other than BD, or personality disorders. The control group consisted of 30 individuals matched for age, sex, BMI, smoking habits, and sociodemographic factors. The controls did not manifest any symptoms of a mental disorder at the time of the study. They were also somatically healthy.

### Clinical assessment

All patients underwent a standard psychiatric, physical and neurological examination. Demographics and family history were collected in the form of a standardized medical history. Presence of psychiatric disorders other than BD was excluded using the Mini International Neuropsychiatric Interview (MINI) questionnaire (55). The patient group was divided into two subgroups according to the type of the disease: BD type I (BD-I) and BD type II (BD-II) (56). To assess the severity of mood disorders, we used the Montgomery-Asberg Depression Rating Scale (MADRS) (57) and the Young Mania Rating Scale (YMRS) (58). Data on previous treatment was based on medical records and the interview. All patients were treated in line with the Polish standards of pharmacological treatment of affective disorders (59, 60). At the time of the study, patients were primarily treated with: lamotrigine in doses ranging from 50 to 300 mg/day (12 patients); valproic acid/sodium valproate in doses ranging from 600 to 1,500 mg/day (10 patients); quetiapine in doses from 100 to 700 mg/day (14 patients); olanzapine in doses of 5–20 mg/day (5 patients); clozapine in doses of 225 mg/day (1 patient) and aripiprazole (1 patient). In addition, the patients were receiving: sertraline (2 patients); venlafaxine (2 patients); escitalopram (1 patient); citalopram (1 patient); mirtazapine (1 patient); paroxetine (1 patient); clomipramine (1 patient); perazine (1 patient); levomepromazine (1 patient) and chlorprothixene (1 patient). For statistical analysis, all doses of antypsychitics were converted to chlorpromazine equivalents (61–63). The drug and its dosage were determined by the patient's physician. The research team did not modify the prescribed treatment in any way. Healthy controls underwent similar examinations to exclude mental disorders and somatic diseases.

### Measurement of complement cascade components

Venous blood samples were collected between 8 am and 9 am after overnight fasting. For determination of the C5b-9 (MAC) levels, we used the Human C5b-9 ELISA Set (BD OptEIA). The levels of C3a and C5a were determined using the Human C3a ELISA Kit and the Human C5a ELISA Kit (BD OptEIA).

### Statistics

The results were analyzed using the STATISTICA 13.1 software (StatSoft, Inc.). In order to verify normality of data distribution, we used the Shapiro-Wilk test. Due to the fact that the parameters were not normally distributed, bivariate analyses were performed using the Mann-Whitney *U*-test. In the next step, the analysis of co-variance (ANCOVA) testing for differences in the levels of complement cascade components was performed. The following variables were used as co-variates: chlorpromazine equivalent dosage, valproate/valproic acid dosage, lamotrigine dosage, BMI and cigarette smoking status. The distribution of C3a and C5a levels fell within acceptable ranges of skewness (C3a: −0.492, C5a: 1.554) and kurtosis (C3a: −0.651, C5a: 1.834 C5a) and thus this data was not transformed before ANCOVA. The distribution of C5b-9 fell originally beyond acceptable range and it was square root transformed. After data transformation, skewness and kurtosis appeared to be acceptable (skewness: −0.455, kurtosis −0.638) (64). To evaluate correlations between continuous variables, we used the Spearman's rank correlation coefficient. Results were considered significant if the *p*-value was below 0.05.

## Results

General characteristics of patients and controls are shown in Table 1. Both groups did not differ significantly in terms of socio-demographic characteristics, except for vocational status. Indeed, there were significantly more employed individuals in the group of controls. Concentrations of all investigated complement components were higher in the group of patients compared to the control group (Table 2). These differences were also significant in separate analyses of BD-I and BD-II patients. However, BD-II patients presented higher C5b-9 concentrations than BD-I patients (trend level significance).

**Table 1:** Clinical and demographic characteristics of the study sample.

	**Patient group (BDG)** ***n* = 30**	**Control group (CG)** ***n* = 30**	***p***
Age in years (mean ± SD)	48.08 ± 11.54	43.90 ± 10.74	0.0699
Sex	Male 15 (50.0%)	Male 13 (43.3%)	0.6650
	Female 15 (50.0%)	Female 17 (56.7%)
Marital status	Single 4 (13.3%)	Single 5 (22.7%)	0.2359
	Married 17 (56.7%)	Married 13 (59.1%)
	Partnership 2 (6.7%)	Partnership 3 (13.6%)
	Widow(er) 1 (3.3%)	Widow(er) 0 (0.0%)
	Divorced 6 (20.0%)	Divorced 1 (4.6%)
Education	Elementary 0 (0.0%)	Elementary 0 (0.0%)	0.3083
	Vocational 5 (16.7%)	Vocational 1 (4.5%)
	Secondary 11 (36.7%)	Secondary 8 (36.4%)
	Higher 14 (46.6%)	Higher 13 (59.1%)
Occupation	Student 1 (3.3%)	Student 4 (18.2%)	0.0012
	Employed 15 (50.0%)	Employed 18 (81.8%)
	Unemployed 2 (6.67%)	Unemployed 0 (0.0%)
	Retired 2 (6.67%)	Retired 0 (0.0%)
	Disability pensioner 10 (33.3%)	Disability pensioner 0 (0.0%)
Residence	Rural 3 (10.0%)	Rural 3 (13.6%)	0.9042
	Small town 1 (3.3%)	Small town 1 (4.6%)
	Medium-size town 4 (13.3%)	Medium-size town 1 (4.6%)
	City 22 (73.4%)	City 17 (77.2%)
Smoking	Yes 13 (43.3%)	Yes 9 (40.9%)	0.8905
	No 17 (65.7%)	No 13 (59.1%)
BMI (mean ± SD)	26.53 ± 4.86	25.15 ± 4.48	0.2173
BD type	BD-I 22 (73.3%)	
	BD-II 8 (26.7%)	
Disease duration (years) (mean ± SD)	17.63 ± 8.22	
Treatment duration (years) (mean ± SD)	11.84 ± 8.73	
MADRS mean score (mean ± SD)	0.07 ± 1.71	
YMRS mean score (mean ± SD)	0.93 ± 1.26	

**Table 2:** Concentrations of complement components in the study sample.

**A. COMPARISONS of C3a, C5a and C5b-9 SERUM CONCENTRATIONS IN BD PATIENTS AND HEALTHY CONTROLS**			
Complement component	BD patient group (*n* = 30)	Control group (*n* = 30)	*p*
C3a [ng/ml]	968.62 ± 82.65	638.78 ± 146.96	0.000001
C5a [ng/ml]	203.56 ± 128.56	74.00 ± 63.01	0.000001
C5b-9 [ng/ml]	408.94 ± 4020.96	267.78 ± 303.48	0.0002
**B. COMPARISONS OF C3a, C5a AND C5b-9 SERUM CONCENTRATIONS IN BD TYPE I PATIENTS AND HEALTHY CONTROLS**			
Complement component	BD type I patient group (*n* = 22)	Control group (*n* = 30)	*p*
C3a [ng/ml]	959.36 ± 67.20	638.78 ± 146.96	0.000001
C5a [ng/ml]	191.49 ± 117.65	74.00 ± 63.01	0.000001
C5b-9 [ng/ml]	369.21 ± 147.31	267.78 ± 303.48	0.0016
**C. COMPARISONS OF C3a, C5a AND C5b-9 SERUM CONCENTRATIONS IN BD TYPE II PATIENTS and HEALTHY CONTROLS**			
Complement component	BD type II patient group (*n* = 8)	Control group (*n* = 30)	*p*
C3a [ng/ml]	994.08 ± 117.26	638.78 ± 146.96	0.000001
C5a [ng/ml]	236.75 ± 158.86	74.00 ± 63.01	0.0001
C5b-9 [ng/ml]	518.18 ± 149.58	267.78 ± 303.48	0.0007
**D. COMPARISONS of C3a, C5a and C5b-9 SERUM CONCENTRATIONS IN BD TYPE I PATIENTS AND BD TYPE II PATIENTS**			
plasma factor	BD type I patient group (*n* = 22)	BD type II patient group (*n* = 8)	*p*
C3a [ng/ml]	959.36 ± 67.20	994.08 ± 117.26	0.0575
C5a [ng/ml]	191.49 ± 117.65	236.75 ± 158.86	0.6559
C5b-9 [ng/ml]	369.21 ± 147.31	518.18 ± 149.58	0.0292

The ANCOVA revealed that there were significant effects of BD diagnosis on the levels of C3a and C5a after co-varying for BMI, smoking, chlorpromazine equivalent dosage and mood stablizer dosage (Table 3). However, differences in the levels of C5b-9 appeared to be insignificant after controlling for the same co-variates.

**Table 3:** The ANCOVA results testing for differences in the levels of complement cascade components after co-varying for potential confounding factors.

	**BD**		**Chlorpromazine equivalent**		**Valproate/valproic acid**		**Lamotrigine**		**BMI**		**Smoking**	
	***F***	***p***	***F***	***p***	***F***	***p***	***F***	***p***	***F***	***p***	***F***	***p***
C3a	30.032	0.000	0.000	0.993	0.000	0.990	0.014	0.907	0.054	0.817	0.043	0.837
C5a	11.112	0.002	1.704	0.199	0.031	0.861	3.306	0.076	0.838	0.365	1.440	0.237
sqrtC5b9	4.022	0.051	0.117	0.734	0.021	0.885	0.036	0.850	0.001	0.975	0.030	0.864

There were no significant correlations between the concentrations of complement cascade components and the scores of MADRS and YMRS (Table 4).

**Table 4:** Correlation between YMRS score, MADRS score and C3a, C5a, C5b-9 concentrations in the study sample.

**Complement component**	**MADRS**			**YMRS**		
	**R_s_**	**${\text{R}}_{\text{s}}^{2}$**	**p**	**R_s_**	**${\text{R}}_{\text{s}}^{2}$**	**p**
C3a	−0.0918	0.0084	0.6296	0.0936	0.0088	0.226
C5a	−0.1926	0.0371	0.3079	−0.0326	0.0011	0.8642
C5b-9	0.2703	0.0731	0.1486	0.1520	0.0231	0.4226

## Discussion

We demonstrated significantly higher concentrations of each of the three identified complement cascade components in patients with BD compared to healthy subjects. Such patterns were also observed when comparing the distinguished BD-I and BD-II patient groups to the control group. When the concentrations were compared between BD-I and BD-II patients, there was a higher C5b-9 concentration in patients with BD-II subtype. However, due to the small number of BD-II patients, the latter difference should be interpreted with caution. Despite elevation of levels of all examinated components, only C3a and C5a were elevated in BD patients after adjustment for potential confounding factor related to differences in BMI and cigarette smoking as well as medication effects. Differences in the levels of C5b-9 appeared to be insignificant after controlling for potential confounders.

It is difficult to refer our results to current evidence in the field as there is a scarcity of srudies addressing these alterations. For instance, Spivak et al. found no significant differences in the concentrations of C3 and C4 between BD patients and patients with other psychiatric disorders or healthy controls in study on forty individuals, of whom eight were diagnosed with BD. However, the authors did not test the concentrations of the active forms of complement cascade components (65). Another study revealed that serum concentrations of C3 and C4 in patients with BD were comparable to the levels in healthy individuals, but significantly lower than in schizophrenia patients (66). Active forms of complement cascade components were also not measured in this study. In turn, there are reports on higher peripheral concentrations of C3, C4, and C6 in the course of a manic episode in BD patients as compared to healthy subjects (67). Similarly, higher concentrations of C4 were earlier observed in patients during the course of bipolar psychosis (68).

Reports on the plasma concentrations of complement components in other psychiatric disorders are also scarce. Studies on mobilization of stem cells into the peripheral blood in patients with psychotic disorders also involved evaluation of components of the complement cascade, as potential factors influencing the process of mobilization of these cells. They showed reduced C3a concentrations in untreated patients with first-episode psychosis. After the initiation of antipsychotic treatment, there was no significant difference in C3a levels as compared to the control group. A second similar study on patients with panic disorder showed decreased levels of all investigated components, namely C3a, C5a, and C5b-9 (69–71). On the other hand, in a study examining concentrations of C4 and sC5b-9 (soluble C5b-9), Akcan et al. (72) found lower concentrations of these components in patients with chronic BD, both in the acute and chronic phase, compared to healthy controls and individuals with the first episode of mood disorders in the course of BD. These concentrations were inversely correlated with the YMRS score and duration of the disease. We did not observe any correlation between the levels of complement cascade components with YMRS and MADRS scores. It should be noted, however, that in contrast to our study, Akcan et al. examined patients in the period of unstable mood and treated with, among others, salts of lithium, which was a clear difference between theirs and our study (72).

Due to their ability to activate and attract leukocytes, anaphylatoxins C3a and C5a seem to be an important link in the inflammatory processes associated with the complement system within the CNS. Importantly, C3a is also a key element in the endothelial and leukocyte activation within the CNS (73). Although in certain situations it may have anti-inflammatory effects, its pro-inflammatory effects take over in the course of chronic reactions, affecting progression of the disease (74, 75). Thus, it seems that the increased concentrations of the complement cascade components observed in the present study may be indicative of a chronic inflammatory process, which could give rise to neurodegenerative changes in the CNS (76).

As previously mentioned, in healthy individuals the blood-brain barrier (BBB) prevents the entry of proteins, including the complement, into plasma. However, pathological conditions may lead to the blood-brain barrier leakage, affected by, among others, C5a. It has been shown that after several hours from such a leakage, complement components may penetrate the brain tissue. It is believed that the permeability of the BBB can further progress in with subsequent BD exacerbations (39, 73, 77, 78). For these reasons, increased concentrations of C3a and C5a in patients with BD may significantly affect the central nervous system, including the regenerative processes.

In our study, we demonstrated that there is an activation of the immune system manifested by an increase in the concentrations of C3a and C5a complement cascade components in the course of BD. However, it should be clearly stated here that the limitation of the study was a small sample size. In their aforementioned study, Kucharska et al. demonstrated a lower C3a concentration in patients with first-episode psychosis as compared to healthy controls (69). In agreement with these findings, complement cascade components have been found to be helpful in differentiating first-episode psychosis (79). In the study on panic disorder, on the other hand, there was no evidence of increased complement activation (70). Therefore, it seems that complement system alterations might be different in distinct groups of mental disorders. Still, there are no reports on C3a, C5a, and C5b-9 levels in depressive disorders or in early stages of BD, i.e., situations involving greatest diagnostic difficulties (71).

In summary, BD patients experience a systemic dysfunction of the immune system manifested by the activation of the complement cascade. In the future, components of the complement system may become useful in research of a biomarker helpful in diagnosing BD. However, further studies addressing complement cascade alterations in various stages of BD and other mental disorders are needed to establish their relevance as potential biomarkers.

## Ethics statement

All subjects gave written informed consent in accordance with the Declaration of Helsinki. The protocol was approved by the by the Bioethical Committee of the Pomeranian Medical University, at its meeting of 10.29.2014 r., resolution No KB-0012/127/12, supplemented with consents KB-0012/70/14 of 10.13.2014 and KB 0012/48/15 of 03.23.2015.

## Author contributions

AR: Patients recruitment, investigation, methodology, validation, writing–original draft, writing–final version. MJ: Patients recruitment, investigation. MB: Investigation. BD: Investigation and methodology. LS: Investigation and project administration. BM: Methodology and revision–original draft. MR: Methodology, supervision, and validation. JR: Investigation and methodology. JS: Investigation, methodology, project administration, supervision, and validation. JK-M: Patients recruitment, investigation, methodology, supervision, validation, writing–original draft, writing–final version.

### Conflict of interest statement

The authors declare that the research was conducted in the absence of any commercial or financial relationships that could be construed as a potential conflict of interest.
